# Dexmedetomidine attenuates postoperative spatial memory impairment after surgery by reducing cytochrome C

**DOI:** 10.1186/s12871-023-02035-x

**Published:** 2023-03-20

**Authors:** Lina Sun, Kun Niu, Jian Guo, Jingru Tu, Baofeng Ma, Jianxiong An

**Affiliations:** 1 School of Anesthesiology, Weifang Medical University, No. 7166, Baotong West Street, Weicheng District, Weifang, Shandong 261000 China; 2 Department of Anesthesiology, Pain& Sleep Medicine, Affiliated Hospital of Weifang Medical University, Shandong, China; 3 Department of Anesthesiology, Pain & Sleep Medicine, Medical University &Beijing Institute of Translational Medicine, Aviation General Hospital of China, Chinese Academy of Sciences, Beijing, China; 4 Savaid Medical School, University of Chinese Academy of Sciences, Beijing, China

**Keywords:** Dexmedetomidine, Anesthesia and surgery, Mitochondrial damage, Cognitive impairment, Hippocampus

## Abstract

**Background:**

Anesthesia and surgery can induce perioperative neurocognitive disorders (PND). Mitochondrial dysfunction has been proposed to be one of the earliest triggering events in surgery-induced neuronal damage. Dexmedetomidine has been demonstrated to attenuate the impairment of cognition in aged rats induced by surgery in our previous study.

**Methods:**

Male Sprague-Dawley rats underwent hepatic apex resection under anesthesia with propofol to clinically mimic human abdominal surgery. The rats were divided into three groups: Control group, Model group and Dexmedetomidine (Dex) group. Cognitive function was evaluated with the Morris water maze (MWM), Open Field Test (OFT)and Novel object recognition task (NOR). Ultrastructural change in neuronal mitochondria was measured by transmission electron microscopy. Mitochondrial function was measured by mitochondrial membrane potential and activities of mitochondrial complexes. Neuronal morphology was observed with H&E staining and the activation of glial cells was observed by immunohistochemistry in the hippocampus. Protein levels were measured by Western blot (WB) and immunofluorescence at 3 and 7 days after surgery.

**Results:**

Surgery-induced cognitive decline lasts three days, but not seven days after surgery in the model group. Transmission electron microscope showed the mitochondrial structure damage in the model group, similar changes were not induced in the Dex group. Dexmedetomidine may reverse the decrease in mitochondrial membrane potential and mitochondrial complex activity. Compared with the Control group, the expression of cytochrome c was significantly increased in model group by Western blot and immunofluorescence on days 3, but not day 7. Rats from the Model group expressed significantly greater levels of Iba-1 and GFAP compared with the Control group and the Dex group.

**Conclusion:**

Dexmedetomidine appears to reverse surgery-induced behavior, mitigate the higher density of Iba-1 and GFAP, reduce the damage of mitochondrial structure and function by alleviating oxidative stress and protect mitochondrial respiratory chain, thus increasing cytochrome c oxidase (COX) expression and downregulate the expression of cytochrome c protein in the hippocampus of rats.

**Supplementary Information:**

The online version contains supplementary material available at 10.1186/s12871-023-02035-x.

## Background

Perioperative neurocognitive disorder (PND) has been found as a common postoperative complication, posing a serious threat to the quality of life of patients, especially the elderly [1]. It is the defined as the decrease in orientation, attention, perception, consciousness, and judgment after surgery [2]. Although PND has been extensively studied, its potential pathogenesis remains unclear due to contradictory results and controversial evidence. Postoperative delirium has been reported in 10–60% of elderly surgical patients, dependent on the surgery performed [3].

The understanding of underlying pathophysiology of PND is increasing, which suggests a prominent role played by neuroinflammation and mitochondrial dysfunction [4]. Mitochondria are the main provider of energy for neurons, and as such, neurons have high vulnerability to death or injury caused by mitochondrial dysfunction. Mitochondrial dysfunction has been increasingly recognized as an essential contributor to PND [5]. Mitochondrial fragmentation during dysfunction is related to mitochondrial osmotic swelling, collapse of the mitochondrial membrane potential (Δ_Ψm_) and dysfunction of the electron transport chain [6]. Existing studies have suggested that mitochondrial imbalance is one of the earliest triggering events in neurodegenerative diseases [7].

Our previous study suggested that deep anesthesia is related to a lower incidence of PND in patients who underwent microvascular decompression under propofol total intravenous anesthesia (TIVA) [3, 8–10]. A PND rat model induced by hepatectomy during propofol TIVA was established to investigate the potential mechanism of deeper anesthesia protection cognition. The results revealed that deep anesthesia can reduce the damage of spatial learning and memory by maintaining cytoskeleton [11]. Our previous studies confirmed that mitochondrion-associated pathways play a role in the impairment of postoperative learning and memory caused by surgery and anesthesia. This impairment is also associated with the up-regulated expression of cytochrome c at the molecular level [12], however, the exact mechanism of PND induced by surgery has not been elucidated.

Dexmedetomidine is a highly selective central α-2 adrenergic receptor agonist that produces the effects of sedation, analgesia, antianxiety and sympathetic activity inhibition. Dexmedetomidine is known to reduce the occurrence of delirium, and its anti-inflammatory and neuroprotective effects have been confirmed in animals and clinical trials [3, 13]. Existing studies have suggested that the mechanisms of dexmedetomidine are related to mitochondrial damage [14]. However, the mechanism by which dexmedetomidine reduces the surgery-induced impairment of cognition in aged rats is still unclear. The results of this study suggest that dexmedetomidine is associated with a protective effect on mitochondrial structure and function. This effect is associated with a reduction in oxidative stress and down-regulation of the expression of cytochrome c and cytochrome c oxidase (COX) to prevent spatial learning and memory impairment in rats following surgery and anesthesia.

## Materials and methods

### Animals

All experimental procedures involving animals were approved by the Ethics Committee of Aviation General Hospital (HK2022-02). All animal experiments complied with the ARRIVE guidelines and were performed following the National Institutes of Health.

Guidelines for the Care and Use of Laboratory Animals. Efforts were made to minimize the number of animals used and their suffering. A total of 66 male, 13-14-month-old Sprague-Dawley rats (480–650 g) were used in these experiments and were purchased from Vital River (Beijing, China). After dexmedetomidine administration, survival rate was then recorded for the next 24 h. Based on our and other published data [8, 15, 16], rats in this age range are equivalent to a human aged greater than 45 years, which is considered one condition for the development of memory impairment following surgery. Animals were first acclimatized for a week to a 12/12 h light/dark cycle at temperatures between 20 and 22 °C and humidity of 50 ± 10%, with access to food and water ad libitum. The rats were acclimatized to the laboratory environment for one week before the initiation of experiments.

### Grouping

Fifty-four male Sprague-Dawley rats were randomly assigned to three groups, as follows: Control group (n = 18), Model group (n = 18), and Dex group (n = 18). The rats in the Control group were not given any treatment. The rats in the Model group underwent resection of hepatic apex under propofol based total intravenous anesthetic. Dexmedetomidine (100 µg/mL, Human well Healthcare Group Co., Ltd., Wuhan, China) was dissolved in 0.9% sterile saline and administered at 20 µg/kg intraperitoneally every 2 h for four doses immediately after resection of hepatic apex in the Dex group. The doses of Dexmedetomidine used in this study served to simulate postoperative sedation and are in accordance with a previous report [17].

### Model establishment

**Fig. 1:**
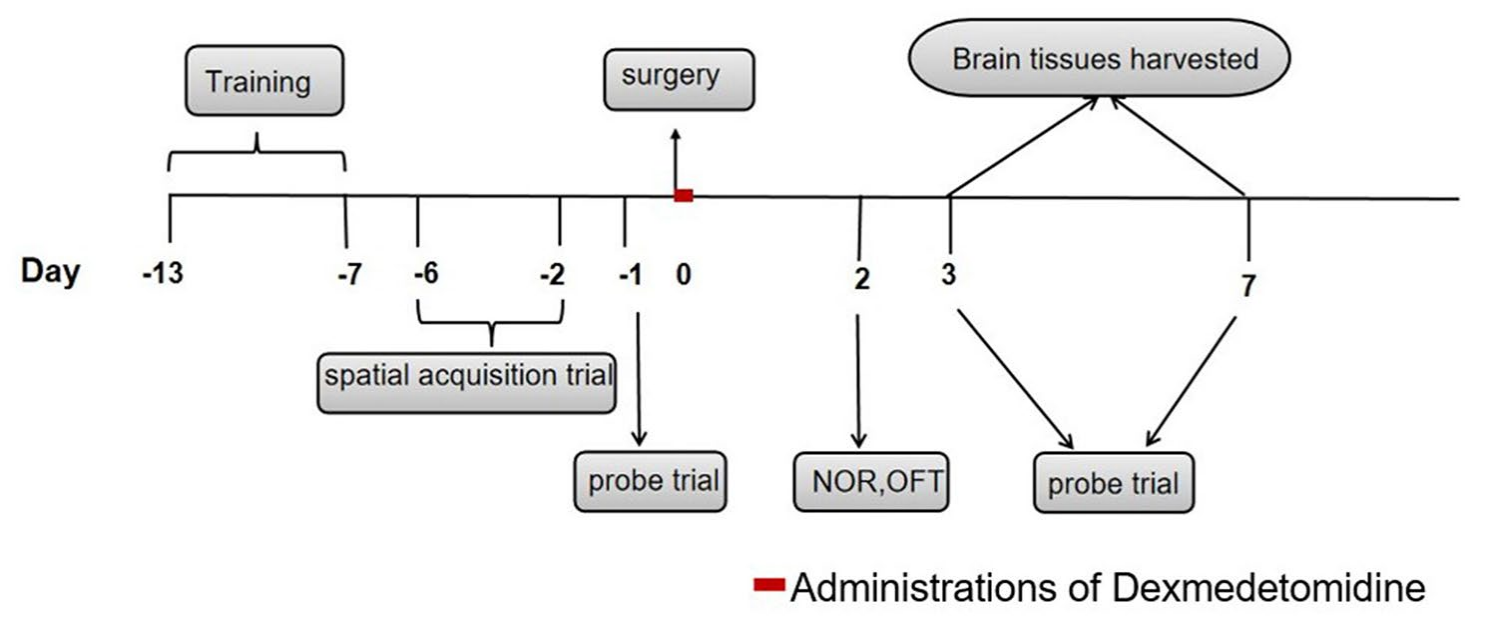
Experimental design. Thirteen-to fourteen-month-old rats were randomly allocated to the Control group (n = 18), the Model group (n = 18), and the Dex group (n = 18). Dexmedetomidine was administered every two hours, for a total of four times after surgery. All rats were trained in the MWM spatial acquisition trial and the probe trial. Open field test and new object recognition tasks were performed on the second day after surgery, mice were sacrificed and brain tissues subsequently harvested at two different time points after surgery

The PND model was induced by the previously described hepatic tip resection [11]. Two rats in the Model group and the Dex group died within 24 h of surgery, but 18 rats in each group were guaranteed to participate in the experiment. In brief, the rats in the Model group and the Dex group were subjected to venous catheterization using vein access under aseptic conditions before anesthesia. The rats then received an induction dose of 12.5 mg/kg intravenous propofol (10 mg/mL, Xi’an Li bang Pharmaceutical& Co., Ltd; Xi’an, Shanxi Province, China), followed by a continuous infusion of 0.625 mg/kg/min for 15 min through a computer-assisted continuous infusion device (Kelly Med™ Syringe Pump, KL-605T, Beijing, China). Analgesia was achieved by the administration of buprenorphine 0.1 mg/kg subcutaneously, immediately after anesthetic induction. After abdominal skin preparation and disinfection, a mid-line incision of 1.5 cm in length was made. The left hepatic duct and artery were ligated, and once ischemic changes were observed in the median and left lateral lobes, these parts were removed. The surgical procedure lasted approximately 5–10 min. Body temperature was maintained at 37 ± 0.5 °C by a heating pad and temperature Control lamp, and all rats have received 0.9% saline solution intravenously to maintain hydration during anesthesia and surgery (Fig. 1).

### Morris Water Maze (MWM)

The MWM was used to assess hippocampal-dependent spatial learning and memory function one week before the surgery and on days 3 and 7 after the surgery [18]. Placing a 10 cm diameter black platform in the pool and fill the pool with water until it is 1 cm higher than the platform. The water is allowed to equilibrate to room temperature (22 °C). The MWM trials include two parts: the spatial acquisition trial and the probe trial. The spatial acquisition trial requires five consecutive days for learning and then subsequent tests of learning.

Briefly, rats are allowed to adjust to the new environment for at least 30 min before testing and then placed into the water from the middle of the four water entry points or quadrants. The time taken for the rats to find the platform is recorded as the escape latency. If the rats fail to find the platform within 60 s, the escape latency is noted as 60 s, the rats are moved to the platform and allowed it to remain there for 30 s. The total training period is 5 days, with training occurring 4 times each day. The average value of the four training periods is used. The probe trial is performed on the 6th day following the last day of training and again 3 and 7 days after surgery. The hidden platform is removed before the probe trial and rats from each group are placed into the water from the opposite side of the original platform. The mice are required to search for the platform from memory, and a camera device is used to record the times of rats crossing over the original platform location as well as the residence time in the target quadrant within 60 s.

### Open Field Test (OFT)

OFT was employed to examine if surgery-induced cognitive impairment was caused by changes in spontaneous motor activity. OFT was conducted in accordance to a previous report [19]. The OFT was carried out in an open field device (50 cm × 50 cm × 37 cm), which consists of Plexiglass wall and black floor. Rats in the device were allowed a 5-min acclimation period before the test. An over-head video camera was used to record the activities of mice. The travelling distance, travelling speed, and duration in the open field were analyzed by SuperMaze (Xinruan information technology Co., Ltd, Shanghai, China).

### Novel object recognition (NOR)

NOR make use of the observation that rats have an innate tendency to explore new objects. If the rats fail to remember an object this serves as a test of their learning and memory [20]. The test consists of a training phase, where in animals were given two identical objects (A and A), and a test phase, where in one familiar object (A) was replaced with a novel object (B). Each trial lasted 5 min and required an interval of one hour before moving to the next phase. The exploratory behavior was recorded by the SuperMaze software (Xinruan information technology Co., Ltd, Shanghai, China). The recognition index (RI) = [time spent exploring the novel object /time spent exploring both objects] was calculated.

### Harvesting of Brain tissue

After each space exploration experiment, three rats in each group were anesthetized by intraperitoneal injection of chloral hydrate (dose 300–350 mg /kg). Following this, 200 ml 0.9% normal saline was perfused, and the hippocampus was removed expeditiously for electro microscopic examination, mitochondrial transmembrane potential (Δ_Ψm_) and electron transfer chain enzyme activity. The heart was fixed by 250 ml of 4% formaldehyde, the whole brain was removed and fixed in paraformaldehyde overnight for hematoxylin eosin (HE), immunohistochemical and tissue immunofluorescence. The pathological images were acquired under a Nikon Eclipse E100 microscope (Nikon Corporation, Tokyo, Japan) fitted with a digital camera at a magnification of 40×.

### Transmission Electron Microscopy

Take a 2×2 mm hippocampal tissue that has been observed for ultrastructure and fixed at room temperature with 1% osmotic acid for 2 h. The dehydration of blocks was carried out with upgraded alcohol and acetone series. Next, the blocks were embedded in the epoxy resin at 60 °C for 48 h. Subsequently, ultrathin sections were cut and double stained with 4% uranium acetate and 0.5% lead citrate. Mitochondrial structures of the CA1 and CA3 regions of the hippocampus were observed with an electron microscope (Hitachi ht7800/ht7700). Use Image-pro plus 6.0 to count the number and area of ​​mitochondria.

### Measurement of the mitochondrial transmembrane potential (Δ_Ψm_)

After treatment, 10 µg/mL JC-1 (Molecular Probes, Beyotime Biotech, China) was applied to the rat brain tissue [21], which were then analyzed using enzyme labeled instrument (Spectrum Max M5 microplate reader, molecular devices US) to measure the fluorescence value.

### Electron transfer chain (ETC) enzyme activity assay

The activity of ETC enzymes was determined based on the activity of the specific donor–acceptor oxidoreductase [22]. After ultrasonic fragmentation with mitochondrial complex detection kit(Beyotime Biotech, China)[23], the activities of mitochondrial complex IV and V enzyme was detected with Spectrum Max M5 molecular device. One nmol NADPH per milligram of hippocampal tissue per minute was defined as an enzyme activity unit.

### Oxidative activity evaluation

Oxidative stress indices were examined in the bilateral hippocampus of rats. Reactive oxygen species (ROS) were measured using a DCFH-DA reactive oxygen species assay kit (Beyotime Biotech, China). A Varioskan LUX microplate reader was used to determine super oxide dismutase (SOD) activity by measuring the absorbance at 450 nm (U/mg protein), and ROS activity was determined by measuring the absorbance at 510 nm (nmol/mg protein).

### Immunofluorescence

Hippocampal tissue was fixed with formalin and embedded in paraffin. Immunofluorescent staining was performed after paraffin removal. In brief, the antigen was repaired by microwave heating in EDTA antigen repair buffer (pH 8.0) for 10 to 15 min. The slices were treated with COX6C antibody (Servicebio, Wuhan, China) and Anti-Cytochrome C antibody (Servicebio, Wuhan, China) for 50 min and incubated overnight. The sections were washed in phosphate buffered saline (PBS) and incubated with fluorescent conjugated secondary antibodies for 50 min at room temperature. After cleaning with PBS, the slices were incubated with 40,6-diamino-2-phenylindole (DAPI) staining solution in the dark for 10 min, and then the spontaneous fluorescence quenching agent was added. Finally, the anti-fluorescence quenching sealing agent was used to seal the slices for microscopic examination and photography. The images were observed and collected under the microscope. Cytochrome C expression in hippocampus CA1 and CA3 regions was marked with red fluorescence and cytochrome c oxidase with green fluorescence.

### Hematoxylin and eosin staining

On the 3rd and 7th day after surgery, the morphological changes of hippocampal CA1 and CA3 neurons in each group (3 rats from each experimental group) were examined using HE staining. After the brain tissue was fixed in 4% paraformaldehyde and dehydrated continuously in ethanol. Finally, the slices were dehydrated and compacted with absolute ethanol for Nikon eclipse E100 microscope (Nikon company in Tokyo, Japan).

### Immunohistochemistry

Microglia and astrocytes were immunohistochemically labeled with Iba-1 and GFAP respectively [24]. Paraffin sections were washed with a dewaxing solution, absolute ethanol and ethanol, and then placed in the citric acid antigen repair buffer (pH 6.0) repair box to repair the antigen. They were blocked in 1% normal goat serum for 30 min at room temperature. Immunohistochemical visualization of glial fibrillary acidic protein (GFAP) and Iba-1 was performed with Rabbit anti mouse GFAP antibody (1:1000, Gb11096, Servicebio, Wuhan, China) and Rabbit anti mouse Iba-1 antibody (1:2000, gb13105-1, Servicebio, Wuhan, China). Finally, the slides were dehydrated and sealed with alcohol, absolute ethanol, n-butanol and xylene in turn. A Nikon eclipse E100 microscope (Nikon company in Tokyo, Japan) was used for visualization after drying. Image J analysis software 6.2 (media cybernetics, silver spring, USA) was used for quantification, and the integral optical density (IOD) of positive stained cells in hippocampal CA1 and CA3 regions was analyzed at 40 times magnification.

### Western blot

The contralateral hippocampus of rats was harvested at 3 and 7 days after surgery.

Tissue samples of the hippocampus were homogenized in a 400 µL 1% PMSFRIPA.

lysis buffer (Servicebio, Wuhan, China) using a glass tissue grinder on ice and then protein concentration was determined with a BCA kit (Sigma-Aldrich, USA). Extracted proteins were separately rated by 10% SDS-PAGE and transferred to nitrocellulose membranes, which were blotted with a variety of specific antibodies: Anti-Cytochrome C antibody (Servicebio, Wuhan, China), Anti-ATP5A antibody (Servicebio, Wuhan, China). The Image J analysis system (National Institutes of Health, USA) was used for quantifying band intensities.

### Statistical analysis

A Shapiro-Wilk test was used to analyze the normality of the data, and the unpaired Student-t test was used to evaluate for significant differences between the two groups. Behavioral test parameters were analyzed using repeated measures and one-way analysis of variance (ANOVA) followed by Tukey’s multiple comparison test for post hoc analysis. P value < 0.05 was considered significant. All statistical analysis was performed using SPSS software (IBM Corporation, Armonk, NY, USA).

## Results

### Dexmedetomidine improved spatial memory deficit induced by surgery in the MWM test

**Fig. 2:**
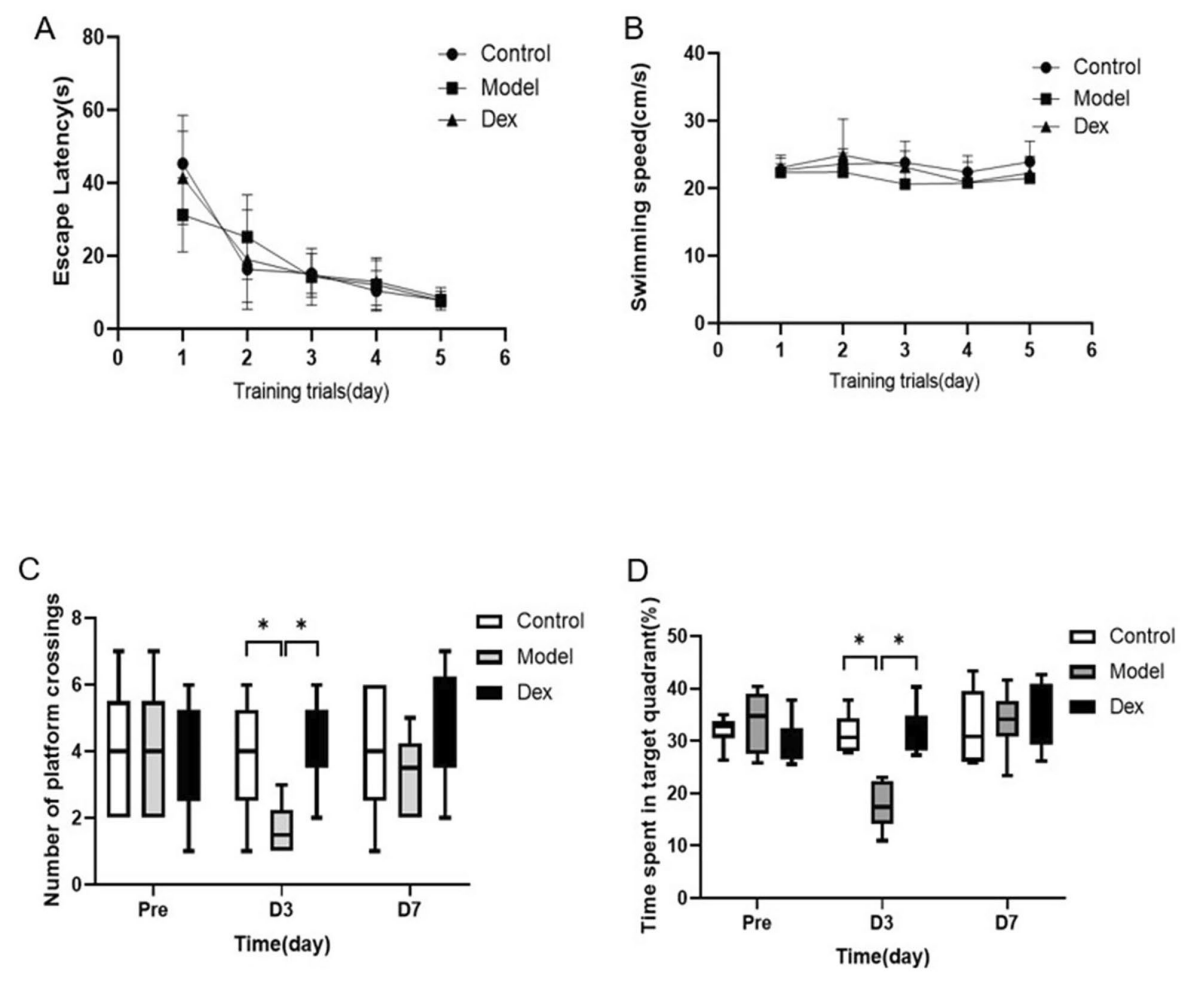
Dexmedetomidine prevents surgery-induced spatial memory deficit in rats tested in MWM. Surgery did not significantly affect escape latency (**A**) or swimming speed (**B**). Rats in the model group presented fewer platform crossings (**C**) and the percentage of time spent in the target quadrant (**D**) at 3 days, but not 7 days after surgery than the Control group. However, the surgery-induced spatial memory deficit was reversed by dexmedetomidine administration (**C** and **D**). N = 18 in the model and Dex group.Data wereanalyzed by repeated measures and one-way ANOVA with Tukey’s multiple comparisons test. Boxplots show median (line) and 25th and 75th percentiles; whiskers show 10th and 90th percentiles. * *P* < 0.05 Compared with Model. MWM, Morris water maze

The effect of Dexmedetomidine on surgery-induced spatial memory impairment was evaluated using the MWM. As depicted in Fig. 2, no significant differences were observed in escape latency (Fig. 2A, P > 0.05) and swimming speed (Fig. 2B, P > 0.05) at each time point among three groups. Compared with the Control group, the model group significantly decreased their platform crossing numbers (Fig. 2C, P < 0.05) at 3 days after surgery. Consistent with the existing report [25],the percentage of time spent in the target quadrant was significantly lower in the model group (Fig. 2D, P < 0.05) at 3 days after surgery, but not day 7 compared with the Control group. The administration of Dexmedetomidine improved the decline in surgery-induced platform crossing number (4.17 ± 1.329 vs. 1.67 ± 0.816 P < 0.001) and shorter time spent in target quadrant (31.73 ± 4.67% vs. 17.65 ± 4.46%; P <0.001) at 3 day after surgery. At 7 days after surgery, there was no significant difference between the groups.

### Dexmedetomidine improved learning and memory deficits induced by surgery in the NOR and OFT tests

**Fig. 3:**
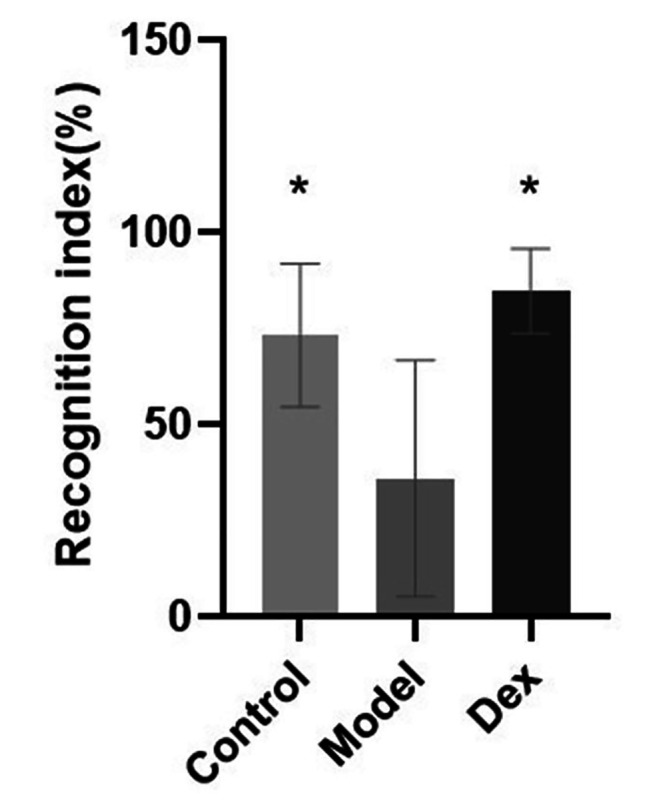
The effect of dexmedetomidine on object recognition ability. Recognition index = [time spent exploring the novel object /time spent exploring both objects] N = 6 per group. Data wereanalyzed by repeated measures and one-way ANOVA with Tukey’s multiple comparisons test. * *P* < 0.05 Compared with Model. NOR, novel object recognition

**Fig. 4:**
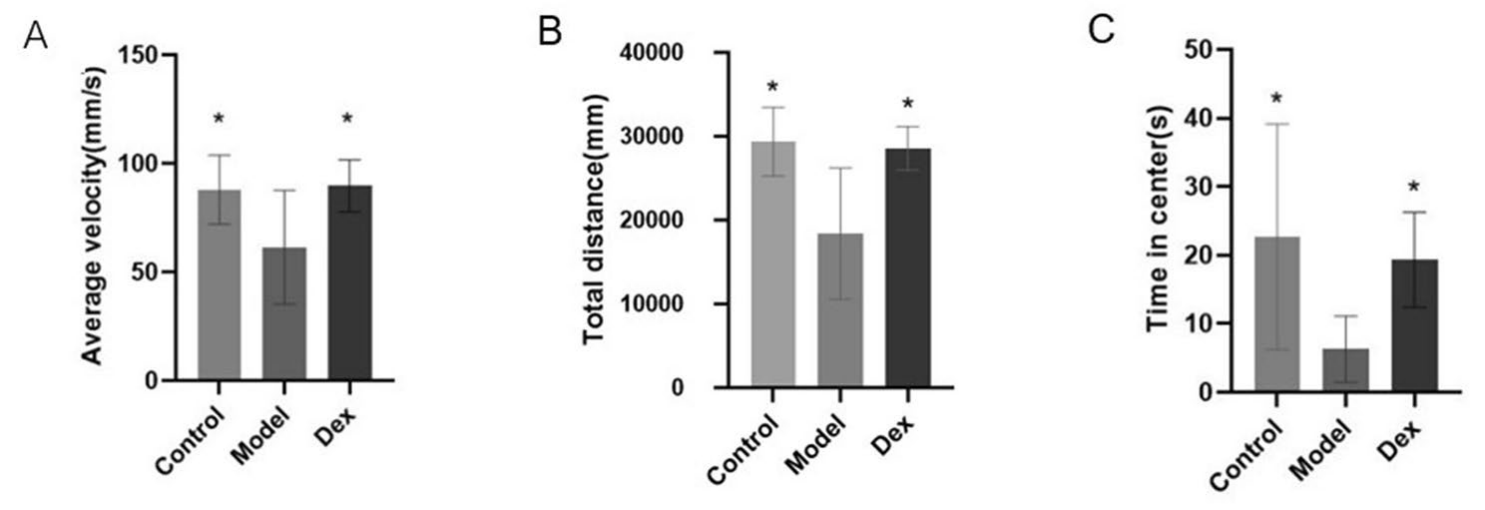
Dexmedetomidine prevents surgery-induced anxiety in rats tested in OFT. (**A**) Average velocity. (**B**) Total distance. (**C**) Time spent in center area. N = 6 per group. Data wereanalyzed by repeated measures and one-way ANOVA with Tukey’s multiple comparisons test. * *P* < 0.05 Compared with Model.OFT, open field test

Learning and memory were evaluated using the novel object recognition and open field tests. Compared with the Control group and the Dex group, the exploration time of new objects in the model group was significantly shortened (Fig. 3, P < 0.05). In the OFT, the average velocity (Fig. 4A), total distance (Fig. 4B) and time in center (Fig. 4C) of the model group was significantly lower than the Control and Dex groups (*P* < 0.05).

### Dexmedetomidine attenuates the hippocampal mitochondrial structure damage induced by surgery

**Fig. 5:**
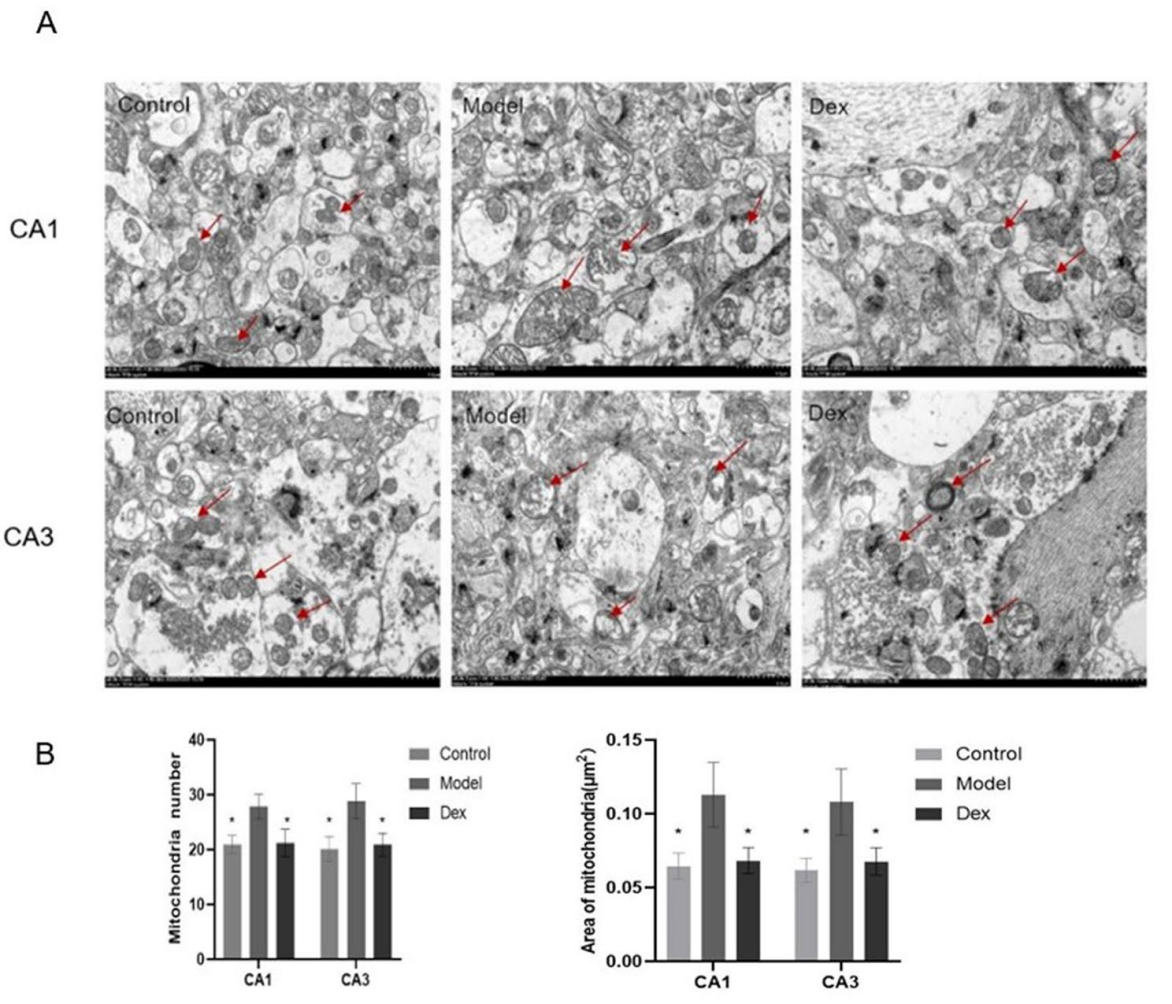
Dexmedetomidine administration prevented surgery-induced mitochondrial structure damage in the CA1 and CA3 areas of hippocampus at 3 days. The hippocampus was harvested after the Morris maze test for observation under microscope. Dexmedetomidine was effective in reducing mitochondrial damage (Fig. 5**A**). Figure 5**B** represents mitochondrial number and area in hippocampus of three groups. Significantly decreased number of mitochondria were found in the hippocampi of Model group. On the contrary, mitochondrial area is significantly increased in the hippocampal tissues from Model group. The objects indicated by red arrows are mitochondria (magnification: × 8,000, scale bar in each panel = 1 μm)

Compared to the Control group, mitochondrial volume increased in the hippocampus CA1 and CA3 regions in the model group. Further, while the model group had a small amount of external membrane of the mitochondria that were healthy, most of the mitochondrial membranes were dissolved and uplifted, with a large number of cristae that were broken and reduced, and the matrix was dissolved and diluted in large sections. The dexmedetomidine group was associated with much less severe mitochondrial damage, including the number of hippocampal mitochondria were more abundant and individual volume was slightly increased. Most mitochondrial membranes were healthy with only a small number of crystals fractured (Fig. 5A). In the model group, the number of mitochondria decreased and the area of ​​mitochondria increased compared with Control group (Fig. 5B).

### Dexmedetomidine ameliorates the decrease in surgery‑Induced membrane potential (Δ_Ψm_) of mitochondria in the rat hippocampus

**Fig. 6:**
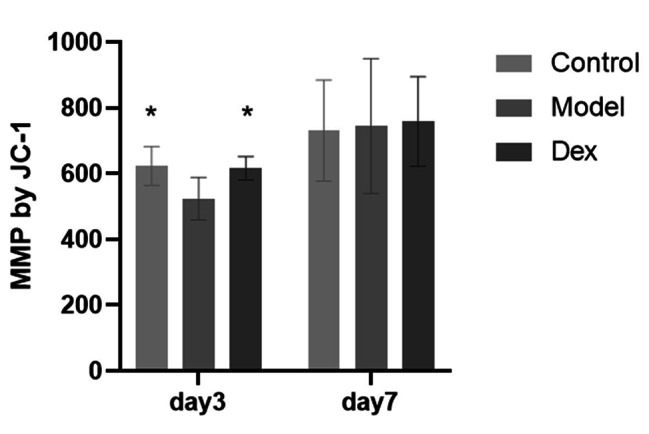
Changes of mitochondrial membrane potential at 3 days and 7 days after surgery in rat hippocampus tissue. Dexmedetomidine alleviated the decrease of mitochondrial membrane potential caused by surgery, and the difference was statistically significant. Data are shown as mean ± SD (N = 6 per group). *P < 0.05 Compared with Model

Mitochondrial membrane potential (MMP) is a vital parameter for evaluating mitochondrial function [26]. As depicted in Fig. 6, JC-1 staining results suggested that surgery may reduce mitochondrial membrane potential, and dexmedetomidine significantly reduces this damage in the hippocampus of rats at 3 days. There was no difference in mitochondrial membrane potential among the three groups at 7 days after surgery.

### Dexmedetomidine increases the activity of mitochondrial electron transfer chain (ETC) complexes in rat hippocampus

**Fig. 7:**
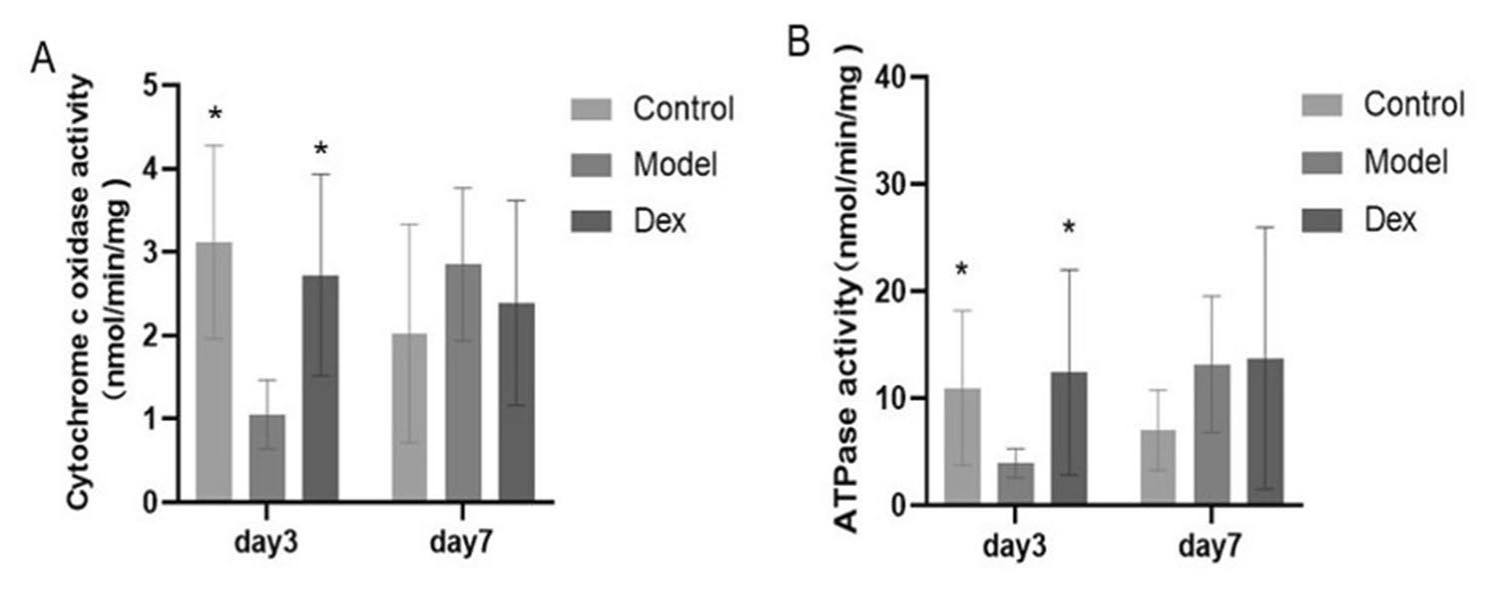
Changes of Cytochrome c oxidase (**A**) and ATP synthase (**B**) activities at 3 and 7 days after surgery in rat hippocampus tissue. Dexmedetomidine increases the activities of ATP synthase and Cytochrome c oxidase in rat hippocampus. Data are shown as mean ± SD (N = 6 per group). * *P* < 0.05 Compared with Model

Mitochondrial complex V, i.e., F_1_F_O_ ATP synthase, catalyzes the synthesis of ATP using energy from an electrochemical proton gradient derived from electron transport [27]. The activities of ATP synthase and cytochrome c oxidase in the model group were reduced at 3 days after surgery in this study, and dexmedetomidine was associated with improvements in this change (Fig. 7P < 0.05).

### Effect of dexmedetomidine treatment on cytochrome c protein and cytochrome c oxidase in CA1 and CA3 expression area of the hippocampus

**Fig. 8:**
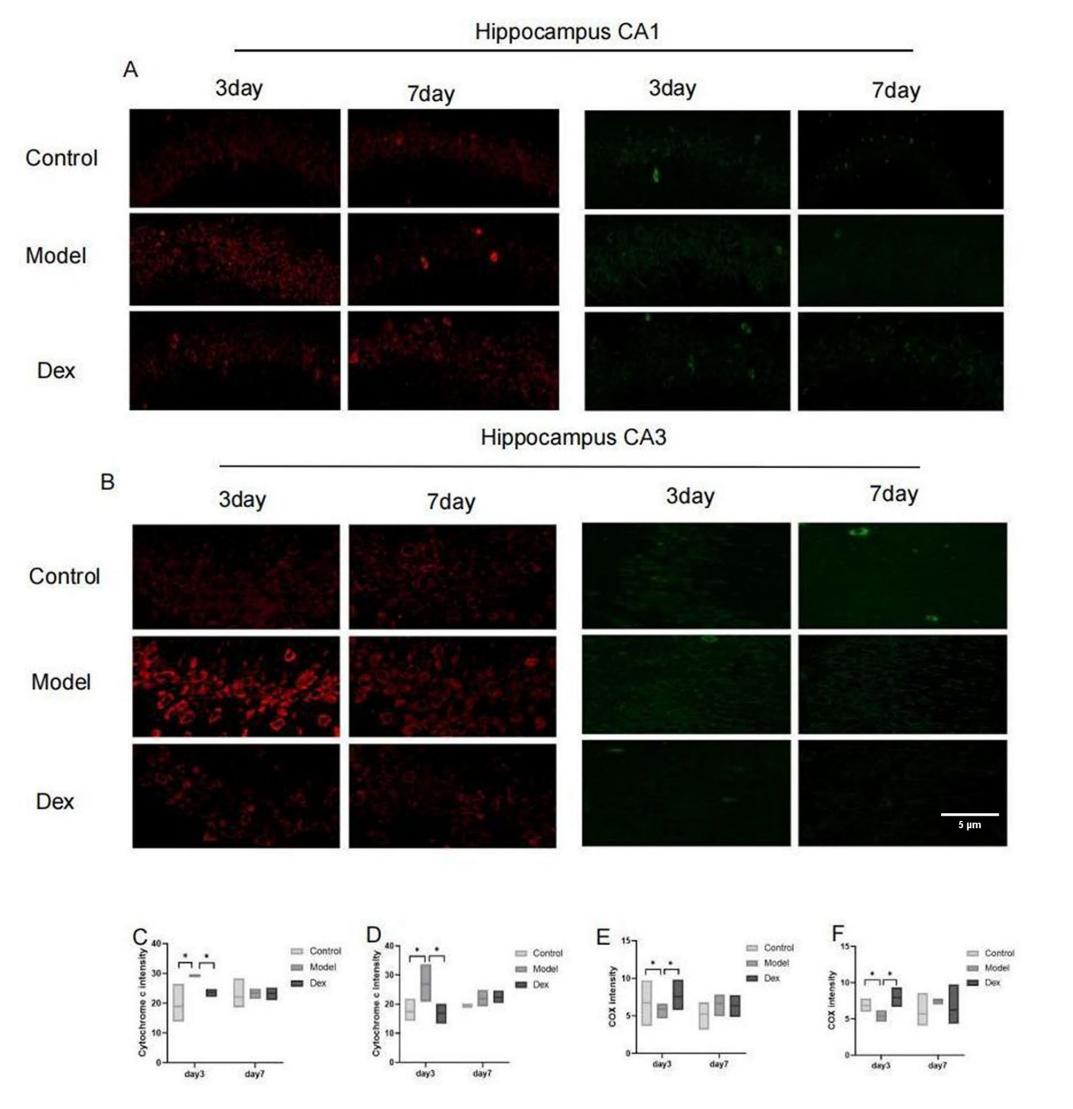
Immunofluorescence staining of hippocampus. (**A**) Representative microphotographs of Cytochrome c protein in CA1and CA3 areas of hippocampal sections from the three experimental groups. Compared to the control group and the Dex group, a higher intensity of the Cytochrome c protein was found in the model group at 3 days, but not 7 days after surgery (at × 63 magnification). (**B**) Representative microphotographs of Cytochrome c oxidase protein in CA1and CA3 areas of hippocampal sections from the three experimental groups. Compared to the Control group and the Dex group, a lower intensity of the Cytochrome c protein oxidase was found in the model group at 3 days, but not 7 days after surgery (at × 63 magnification). (**C**) Cytochrome c protein expression in the CA1 hippocampus of rats. (**D**) Cytochrome c protein expression in the CA3 hippocampus of rats. (**E**) Cytochrome c oxidase expression protein in the CA1 hippocampus of rats (**F**) Cytochrome c oxidase protein expression in the CA3 hippocampus of rats N = 3 per group. Data were analyzed by one-way ANOVA with Tukey’s multiple comparisons test. Scale bar = 20 μm. * *P* < 0.05 Compared with Model

The expression of cytochrome c protein and cytochrome c oxidase was measured by immunofluorescence. Surgical trauma significantly increased cytochrome c protein expression and decreased cytochrome c oxidase in the CA1 and CA3 hippocampus area when examined on postoperative day 3; however, the expression was normalized by day 7 (Fig. 8A and B). Administration of dexmedetomidine down-regulated the levels of cytochrome c protein and up-regulated the levels of cytochrome c oxidase in the hippocampus compared with the model group rats on postoperative day 3 (Fig. 8C F). Hippocampal mitochondrial damage was manifested by the up-regulated expression of cytochrome c and the down-regulated expression of cytochrome c oxidase induced by surgery.

### Dexmedetomidine ameliorated surgery-induced oxidative stress

**Fig. 9:**
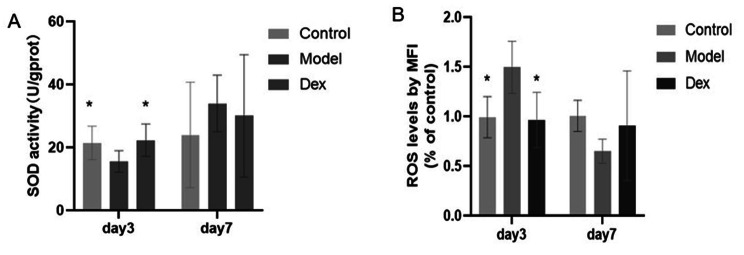
Dexmedetomidine administration ameliorated surgery-induced oxidative stress. (**A**) Activities of superoxide dismutase (SOD) in rat hippocampus tissue (**B**) The content of ROS in rat hippocampus tissue. Data are shown as mean ± SD (N = 6 per group). * *P* < 0.05 Compared with Model

The changes of two critical indicators of oxidative stress were measured, including the content of ROS and activity of SOD, to investigate the effect of Dex treatment on surgery-induced oxidative stress. As depicted in Fig. 9, the level of ROS was significantly reduced associated with dexmedetomidine treatment (1.50 ± 0.262 vs. 0.96 ± 0.278 *P* < 0.05). In contrast, SOD was improved significantly after surgery (15.53 ± 3.399 vs. 21.46 ± 5.334U/g in the Control group, *P* < 0.05). However, dexmedetomidine treatment enhanced SOD activity (15.53 ± 3.399 vs. 22.286 7 ± 5.128 in the Dex group, *P* < 0.05).

### Effect of Dexmedetomidine treatment on pyramidal neurons in the CA1 and CA3 areas of the hippocampus

**Fig. 10:**
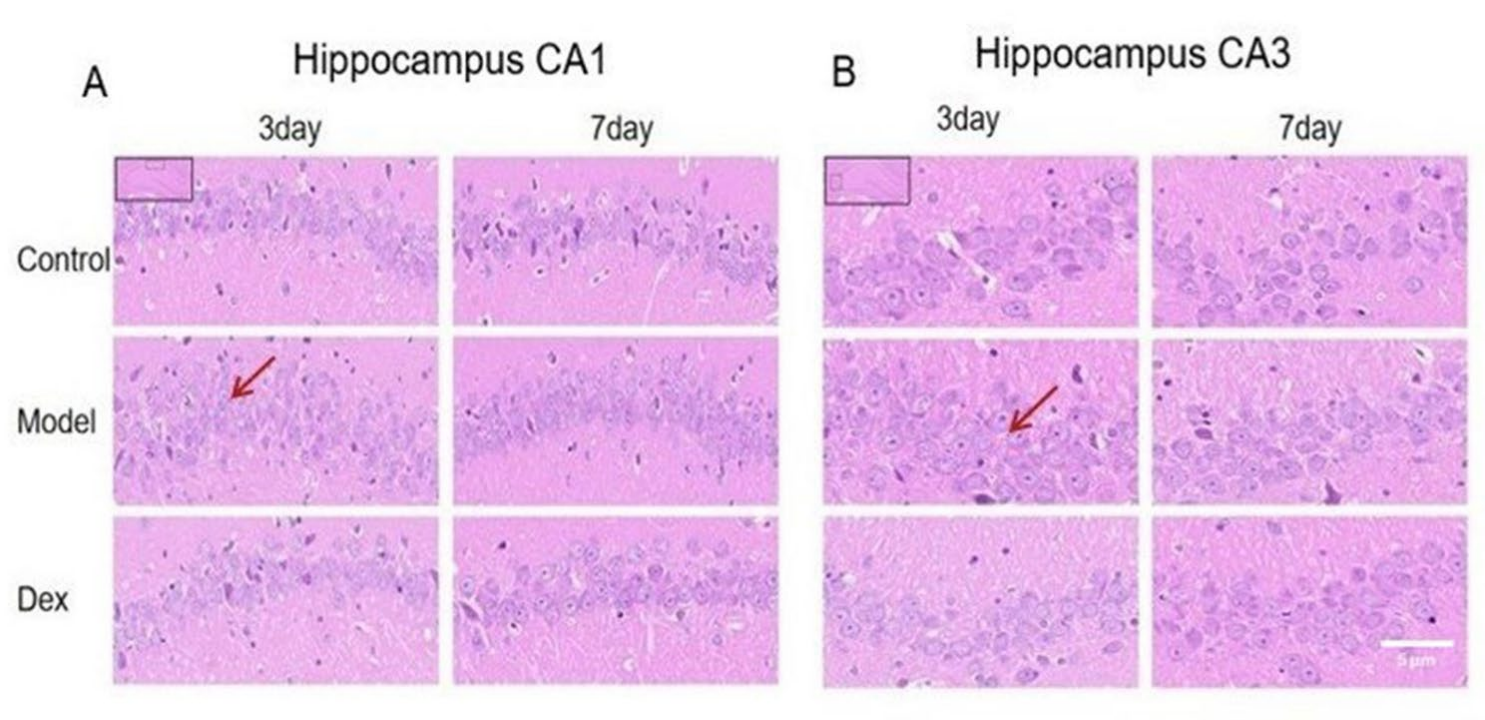
Evaluation of the number of surviving neurons in rats by Hematoxylin-Eosin (HE) staining. (**A**) Representative microphotographs of HE-stained CA1 area of hippocampal sections from the three experimental groups. A higher density of glial cells was found in the model group at 3 days, but not 7 days after surgery, compared to the Control group and the Dex group at × 40 magnification. (**B**) Representative microphotographs of HE-stained CA3 area of hippocampal sections from the three experimental groups. A higher density of glial cells was found in the model group at 3 days, but not 7 days after surgery, compared to the Control group and the Dex group at × 40 magnification. N = 3 per group. Data were analyzed by one-way ANOVA with Tukey’s multiple comparisons test. Scale bar = 20 μm

HE staining was performed to detect neuronal morphology. As depicted in Fig. 10A and B, healthy hippocampal CA1 and CA3 pyramidal neurons were round, and the staining nucleus was significant in the Control group. Compared with the Dex group, the model group was found with an increased density of glial cells, which surrounded the surviving neurons. At 3 days after surgery, a higher density of glial cells was found in the model group than the Control group or the Dex group. The morphological alterations in the CA1 and CA3 hippocampal neurons lasted until at least postoperative day 7.

### Effect of dexmedetomidine treatment on Iba-1 and GFAP expression in CA1 and CA3 areas of the hippocampus

**Fig. 11:**
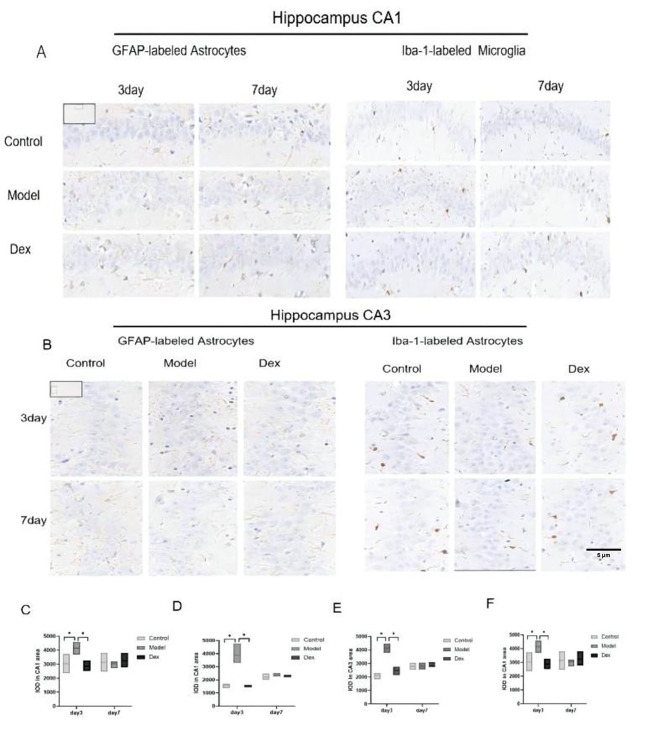
Immunohistochemical staining for Iba-1 and glial fibrillary acidic protein (GFAP) expression in CA1 and CA3 area of hippocampus. (**A**) Representative microphotographs of Iba-1 and GFAP in CA1 area of hippocampal sections from the three experimental groups. Compared to the Control group and the Dex group, a higher density of Iba-1-labeled microglial and GFAP-labeled astrocyte cells were found in the model group at 3 days, but not 7 days after surgery (at × 63 magnification). (**B**) Representative microphotographs of Iba-1and GFAP in CA3 area of hippocampal sections from the three experimental groups. Compared to the Control group and the Dex group, a higher density of Iba-1-labeled microglial and GFAP-labeled astrocyte cells were found in the model group at 3 days, but not 7 days after surgery (at × 63 magnification) (**C**) GFAP expression in the CA1hippocampus of rats. (**D**) Iba-1 expression in the CA1 hippocampus of rats. (**E**) GFAP expression in the CA3 hippocampus of rats. (**F**) Iba-1 expression in the CA3 hippocampus of rats. N = 3 per group. Data were analyzed by one-way ANOVA with Tukey’s multiple comparisons test. Scale bar = 20 μm. * *P* < 0.05 Compared with Model

Hippocampal Iba-1 and GFAP were examined to investigate the activation of microglia and astrocytes after surgery. Surgical trauma significantly up-regulated Iba-1 expression in the CA1 and CA3 hippocampal areas on postoperative day 3. The expression was normalized by day 7 (Fig. 11A). Similar findings were seen with GFAP (Fig. 11B). Administration of dexmedetomidine decreased the levels of GFAP in the hippocampus compared with the model group rats on postoperative day 3 (Fig. 11C and D). A similar down-regulation in IBA-1 levels was also found in the Dex group rats on postoperative day 3 (Fig. 11E F).

### Dexmedetomidine reverses surgery-induced upregulation of cytochrome c expression but not ATPase

**Fig. 12:**
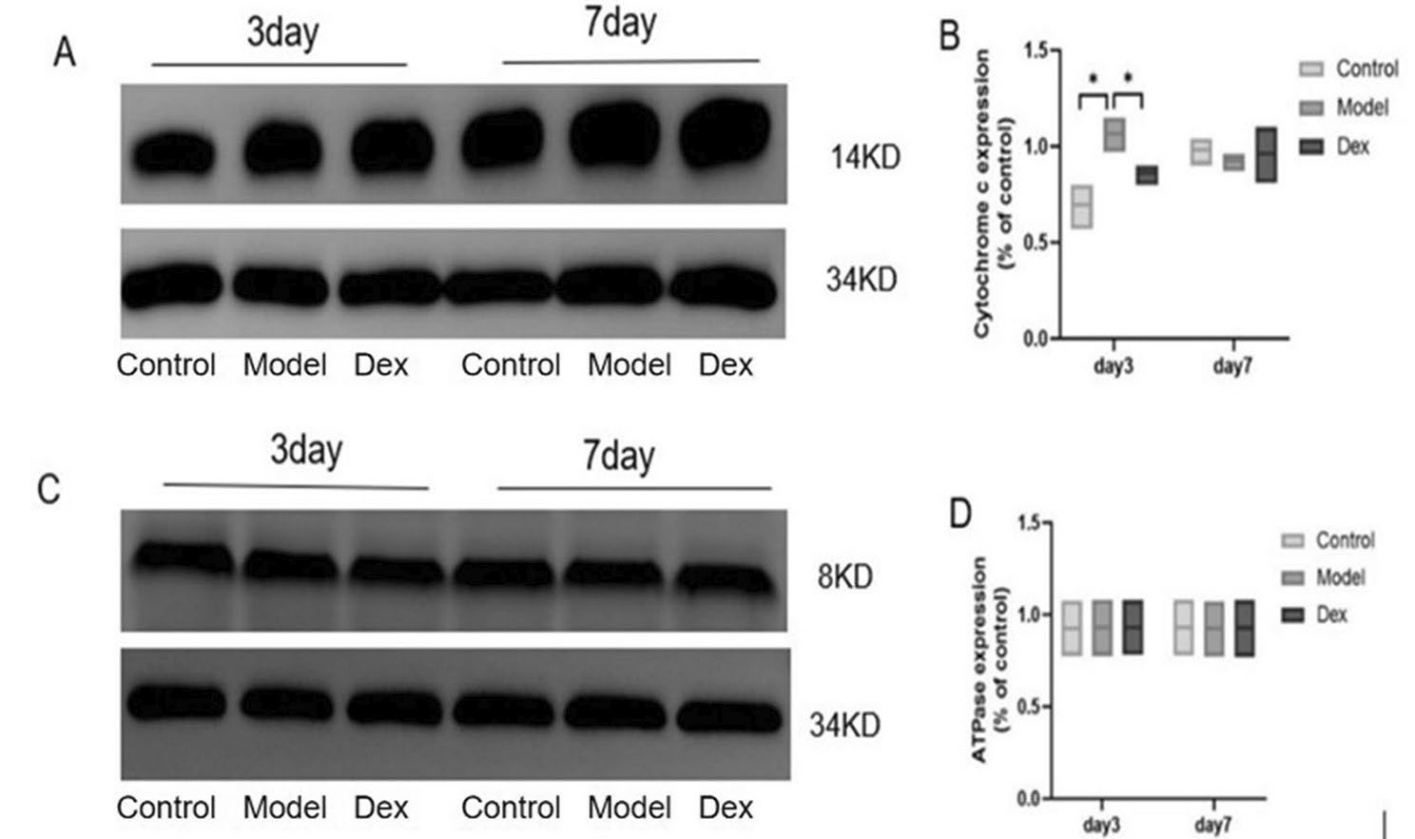
Dexmedetomidine administration prevented surgery-induced upregulation of mitochondrial electron transport chain-related proteins in the hippocampus (**A**) Western blotting showed the expression of Cytochrome c in the hippocampus 3 and 7 days after surgery. (**B**) Quantitative analysis revealed that te expression levels of Cytochrome c in the hippocampus were significantly higher in the model group than in the Control group and the Dex group. (**C**) the expression of ATPase in the hippocampus 3 and 7 days after surgery. (**D**) Quantitative analysis revealed that the expression levels of ATPase in the hippocampus was no difference among the three experimental groups. * *P* < 0.05 Compared with Model

Given the findings that surgery-induced spatial memory deficits are accompanied by mitochondrial damage, cytochrome c was selected for western blot, which is a marker protein of mitochondrial damage. As depicted in Fig. 12, compared with the Control group, cytochrome c expression was significantly up-regulated in the hippocampus on postoperative day 3, but not postoperative day 7 in the Model group. Dexmedetomidine reversed surgery-induced cytochrome c upregulation on postoperative day 3. There was no difference in the expression of ATP synthase among the three groups. (Fig. 12A − 12D).

## Discussion

According to the results of this study, dexmedetomidine is associated with an attenuation in surgery-induced behavioral and memory changes as well as a reduction in mitochondrial damage. In this aged rat model of cognitive impairment caused by surgery, dexmedetomidine may reduce spatial learning and memory impairment. The results of this study, including: structure, function, mitochondrial complex activities, expression of mitochondrion-associated damage proteins, strongly suggest that dexmedetomidine is associated with a reduction in mitochondrial damage induced by surgery.

Existing studies have suggested that mitochondrial damage may play an essential role in cognitive impairment, and it is one of the possible mechanisms of PND [28]. Since the energy supply required for the survival of neurons is dependent on the production of mitochondria; the brain is extremely vulnerable to mitochondrial dysfunction. Mitochondrial dysfunction is one of the earliest triggering events in surgery-induced neuronal damage [7]. Moreover, Previous studies have suggested that mitochondrial dysfunction plays a role in various neurodegenerative disorders (e.g., Alzheimer’s disease, Parkinson’s disease, Huntington’s disease, and amyotrophic lateral sclerosis [29]). The impaired mitochondria can exhibit an altered morphology, decreased membrane potential and disrupted function of the electron transport chain (ETC) complexes: thus these mitochondria take on a crucial significance in the incidence of PND [30].

The findings of this study suggested that mitochondria play an important role in the pathophysiological mechanism of memory impairment induced by surgery. An abnormal mitochondrial morphology strongly suggests mitochondrial damage. Following surgery, the normal mitochondrial structure disappeared, and vacuoles appeared in the hippocampi of cognitively impaired rats. Compared with the Control group, the mitochondrial volume increased in the model group, and most of the mitochondrial membranes were dissolved and uplifted. A considerable number of cristae were broken and fragmented. The findings of this study confirmed that mitochondrial damage is associated with surgery. Functionally, in this study, the mitochondrial membrane potential (Δ_Ψm_) also decreased. Prior studies have suggested that dexmedetomidine can reverse bupivacaine-induced changes in mitochondrial membrane potential [21].

In addition, reduced activity of one or more of the mitochondrial electron transport chain enzymes, or of F_1_F_0_-ATPase (ATP synthase, complex V) can compromise brain ATP synthesis and induce damaging ROS production which if severe, can lead to neuronal death [31]. Mitochondrial ATP synthase emerges as a key hub of cellular functions, controlling the production of ATP, cellular signaling, and fate [32]. ATP synthase activity in the entorhinal cortex of AD patients was significantly lower than that of normal subjects [33]. Our previous results revealed that the overexpression of surf1 mRNA is significantly related to spatial memory defects, and surf1 plays a role in the effective assembly of mitochondrial complex IV (cytochrome c) [34]. Mitochondrial complex IV is the terminal enzyme in the electron transport chain, and plays a crucial role in ATP synthesis. The reduced activity of mitochondrial complex IV has been consistently observed in post-mortem disease samples from Alzheimer’s patients [35]. Consistent with our results, the reduction in activities of mitochondrial complexes IV and V in the model group were reversed by dexmedetomidine.

Interestingly, only ATPase activity changed without the change of expression. The cause may be related to the complexity of the structure and function of ATP synthase. Moreover, existing research suggests that COX activity and mRNA levels of COX subunits I and III decrease in the related cortex from AD patients [36]. Immunofluorescence results showed that the expression of cytochrome c and cytochrome c oxidase were down-regulated in the CA1 and CA3 regions of the hippocampal mitochondria in the model group; however, it was up-regulated in the Dex group. This suggests that cytochrome c oxidase may be a target of dexmedetomidine in the mitochondria. Since impaired COX may lead to the accumulation of reactive oxygen species (ROS) and oxidative stress [37], ROS expression in the hippocampal mitochondria of the model group was up-regulated, and antioxidant SOD activity was reduced in this study. The above change can be reversed by dexmedetomidine. In fact, the increase in oxidative stress and damage to the mitochondrial electrical circuit are interrelated, thus forming a vicious circle [38].

Our previous study suggested that dexmedetomidine down-regulates the expression of Surf1 and cytochrome c protein in the hippocampus in a rat PND model [12]. Cytochrome c is a mitochondrial protein that can be a critical signaling molecule for neuroinflammation as it is released from damaged neurons [39]. Further, this is an important step in the activation of intracellular signaling, resulting in the cascade of caspase activation that leads to apoptotic cell death [40].

Consistent with protein and immunofluorescence results in this study, cytochrome c expression was up-regulated in the hippocampal CA1 and CA3 regions of model rats, which may also explain the mitochondrial damage secondary to protein expression levels. Existing studies have revealed that cytochrome c can cause inflammatory aggregation of microglia or microglia like cells via the toll-like receptor (TLR) 4 [41].

The morphological changes observed in hippocampal neurons with HE staining in the model group had histopathological abnormalities (e.g., glial cell proliferation). The hippocampal activation of microglial cells and astrocytes from postoperative day 3 and day 7 was further observed through immunohistochemical staining. Images showed that compared with the Control group, CA1 and CA3 areas of hippocampus highly expressed Iba-1-labeled microglial cells and GFAP-labeled astrocytes at 3 days, but not 7 days after surgery. It appears that dexmedetomidine administration inhibited the exaggerated microglial cells and astrocytes activation. Imbalance or changes to CNS populations of microglial cells and astrocytes may play a role in many neurodegenerative diseases [42]. Existing reports suggested that the activation of glial cells can lead to subsequent release of proinflammatory mediators, thus leading to mitochondrial and synaptic dysfunction in a wide variety of neurodegenerative disorders [43].

There are several limitations in this study. First, the activity of other mitochondrial respiratory chain enzyme complexes and ATP content were not evaluated. Second, there was no reverse validation with dextromethorphan or protein inhibitors. Third, the control group should be given the same dose of solvent instead of nothing and use 22-month-old rats for a more rigorous experimental design, We will use a larger sample size for molecular validation in future experiments.

## Conclusion

This study provides evidence that dexmedetomidine ameliorates surgery-induced memory impairment and is associated with a reduction in mitochondrial damage in aged rats. This study may have a stimulatory effect on further studies that may elucidate the exact mechanisms of neurotoxicity following surgery and anesthesia and possible targeted interventions for surgery-induced memory impairment.

## Electronic supplementary material

Below is the link to the electronic supplementary material.

Supplementary Material 1

Supplementary Material 2

Supplementary Material 3

Supplementary Material 4

## Data Availability

The raw data supporting the conclusions of this manuscript will be made available by first author, without undue reservation, to any qualified researcher.
