# Supplementary figures and images for: Dexmedetomidine attenuates postoperative spatial memory impairment after surgery by reducing cytochrome C
Source: BMC Anesthesiol. 2023 Mar 20;23:85. doi: 10.1186/s12871-023-02035-x (PMC10026454; doi:10.1186/s12871-023-02035-x)

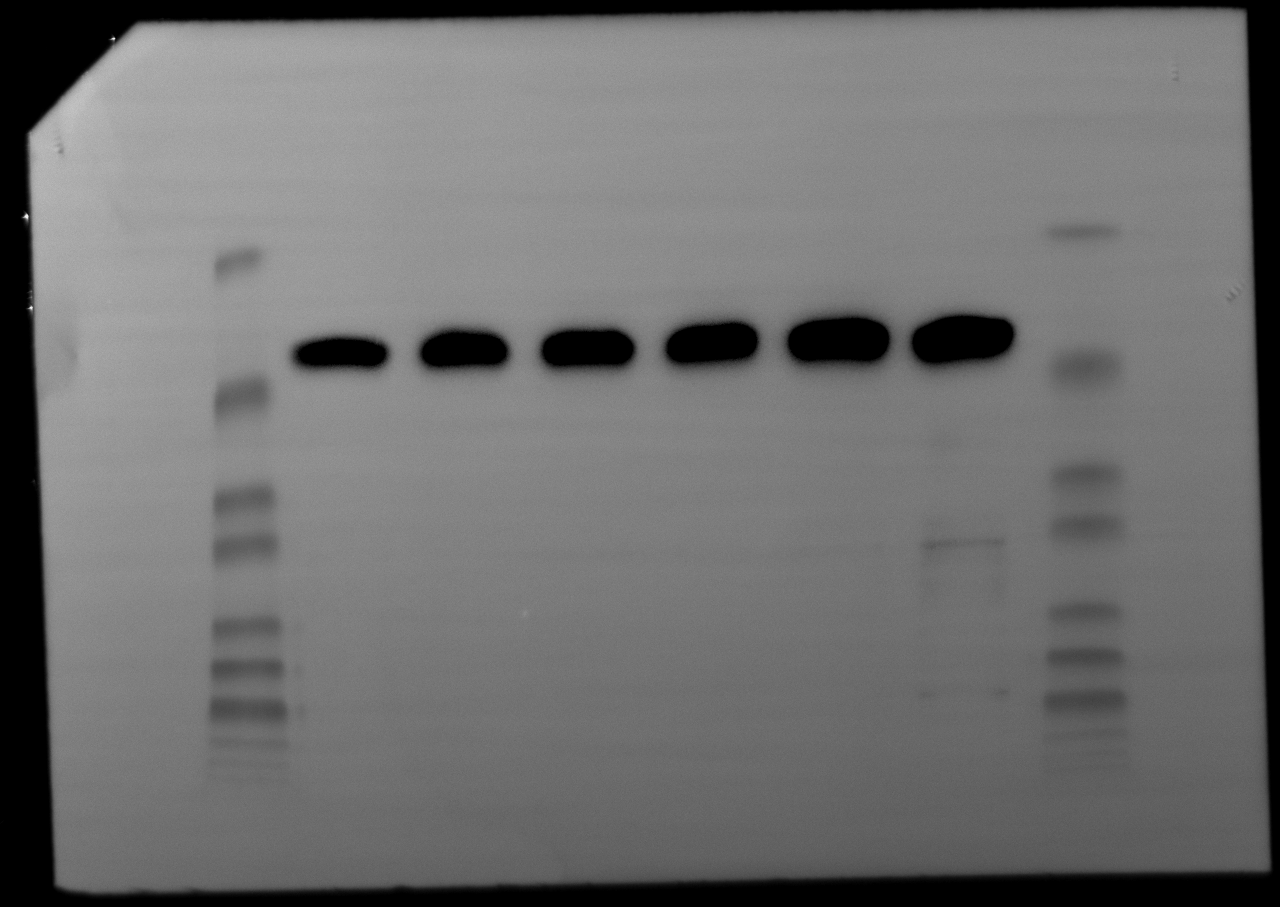

Supplement: Supplementary file 1 — Supplementary Material 1 [file 12871_2023_2035_MOESM1_ESM.tif]

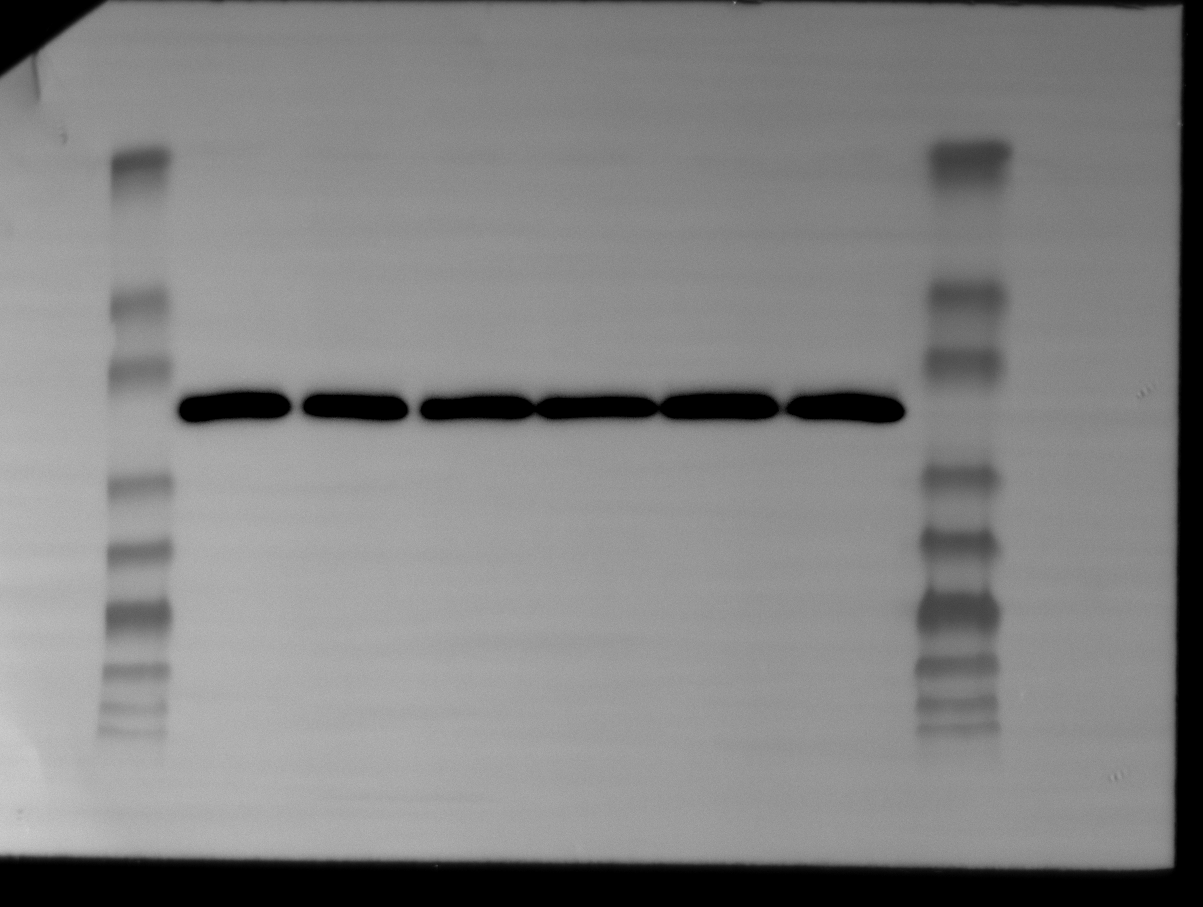

Supplement: Supplementary file 2 — Supplementary Material 2 [file 12871_2023_2035_MOESM2_ESM.tif]

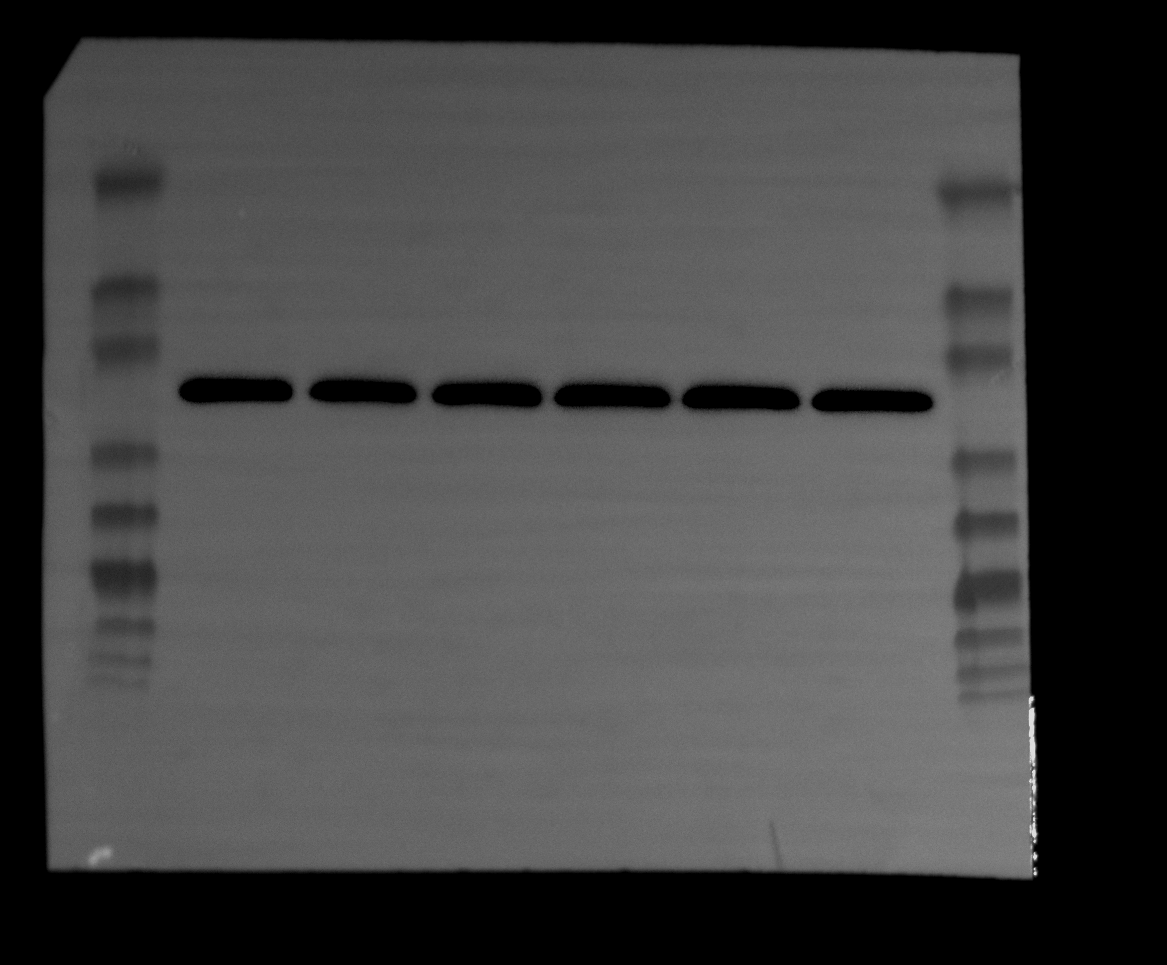

Supplement: Supplementary file 3 — Supplementary Material 3 [file 12871_2023_2035_MOESM3_ESM.tif]

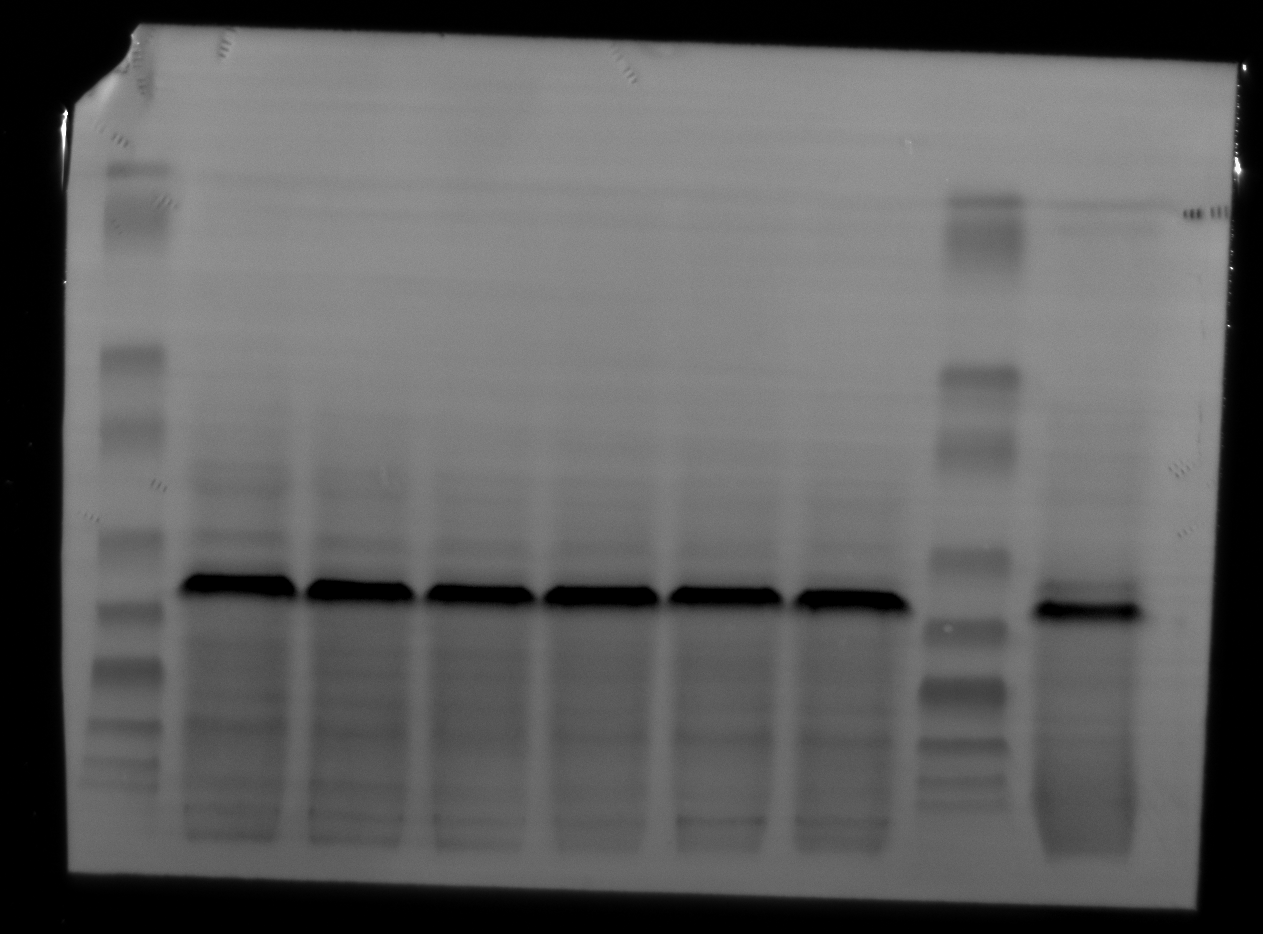

Supplement: Supplementary file 4 — Supplementary Material 4 [file 12871_2023_2035_MOESM4_ESM.tif]
